# Reimagining effective workplace support for health workers 

**DOI:** 10.2471/BLT.24.291896

**Published:** 2024-06-01

**Authors:** Jenny JW Liu, Anthony Nazarov, Patrick Smith, Andrea Phelps, David Forbes, Nicole Sadler, Fardous Hosseiny, Sarah Dougherty, Rosilee Peto, Marion Cooper, Marc Bilodeau, Suzanne Bailey, Jodi Younger, Adam Dukelow, Sandy Jansen, Andrew Davidson, Cara Vaccarino, Karen Monaghan, Sandra Northcott, Linda Mohri, Patricia Hoffer, J Don Richardson

**Affiliations:** a MacDonald Franklin OSI Research and Innovation Centre, Lawson Research Institute, 750 Base Line Road East, Suite 300, London N6C2R5, Ontario, Canada.; b Phoenix Australia, Centre for Posttraumatic Mental Health, Melbourne, Australia.; c Atlas Institute for Veterans and Families, Ottawa, Canada.; d The Royal Ottawa Hospital, Ottawa, Canada.; e St. Joseph’s Health Care London, London, Canada.; f Canadian Armed Forces, Ottawa, Canada.

The coronavirus disease 2019 (COVID-19) pandemic revealed systemic weaknesses in health-care systems worldwide. The breadth of challenges left health workers overwhelmed and overstretched, reducing their professional efficacy and causing long-term issues with retention, recruitment and education of future cohorts.^1^^,^^2^ In response, a rush to repurpose occupational support emerged^3^ However, these efforts have demonstrated limited effect.^3^^–^^5^ We draw on lessons and insights from employee experience, resilience and organizational psychology, and leverage international perspectives on leadership and staff engagement to propose four recommendations towards optimal support of the health workforce. 

First, shift from individual-level to organizational-level resilience. The lack of efficacy in individual-level resilience interventions reflects the understanding that resilience is not an individual endeavour. Contributors to resiliency are multidimensional and multisystemic in nature.^6^ Well-being programmes often place the onus of resilience on the individual. Instead, organizations need to acknowledge their roles and responsibilities in fostering a working environment that is psychologically safe, supportive, and that promotes thriving and growth.

Second, move towards a dual continuum of mental health. Aligning with the World Health Organization’s definition of well-being,^7^ conceptualization of mental health as the absence of mental illness necessitates an evolution to a dual continuum that considers positive mental well-being (that is, flourishing versus languishing).^8^ This approach destigmatizes mental health, normalizes daily challenges in individual lives, and seeks to support those managing mental illness and everyday struggles. Support involves a broader spectrum of interventions that go beyond symptoms reduction, to encompass sustainable approaches that enhance prevention, health promotion and optimization of function, satisfaction and meaning.

Third, engage health workers. The opposite of burnout is not resilience; it is authentic engagement. Health workers cite institutional demoralization, distrust and moral distress as top contributors to burnout.^4^^,^^9^ Generic solutions are insufficient; health workers need to feel engaged in the process of identifying and prioritizing issues, brainstorming and co-developing solutions, and contributing to implementation and evaluation.^10^ Even in the absence of immediate solutions, health workers should feel that their leaders back them and are aware of their needs, such that workers feel seen, heard, understood and valued in the process.^10^

Fourth, recognize that health worker burnout is a systemic, universal issue. The effects of the COVID-19 pandemic have amplified many of the pre-existing challenges, such as health worker recruitment, retention and workplace well-being. Solutions need to depart from single, isolated initiatives towards an emphasis on systems-level implementation and communication of change. Health organizations seeking to improve support must consider the adoption of implementation science frameworks in which the level of uptake, evaluation of effectiveness, barriers and facilitators, and maintenance strategies are incorporated in the short and long term.

In current organizational structures, leaders and individuals in specialized roles are assigned the responsibility to initiate and implement change. However, they often face limitations due to the constraints of their authority and the scope of their mandates. As a result, their efforts may lack autonomy and meaningful impact, leading to workplace cultures that are resistant to change. These four key lessons collectively call for integrated strategies that go beyond traditional, top-down or bottom-up solutions.^9^ Through continued engagement and collaboration, health workers and leaders are collectively tasked to co-identify problems, co-generate solutions, and build agency towards an organizational future with more robust support systems. This non-hierarchical approach is pivotal in transforming how we sustainably address the challenges in our health systems and support those at the forefront of health-care delivery.
